# Unity of heaven and humanity: Mediating role of the relational-interdependent self in the relationship between Confucian values and holistic thinking

**DOI:** 10.3389/fpsyg.2022.958088

**Published:** 2022-09-30

**Authors:** Zhen-Dong Wang, Yi-Meng Wang, Huan Guo, Qian Zhang

**Affiliations:** ^1^School of Basic Medical Sciences, Shanghai University of Traditional Chinese Medicine, Shanghai, China; ^2^School of Psychology, Nanjing Normal University, Nanjing, China; ^3^Institute of Analytical Psychology, City University of Macau, Macao, Macao SAR, China; ^4^School of Human Resources, Guangdong University of Finance & Economics, Guangzhou, China; ^5^Naval Medical Center, Naval Medical University, Shanghai, China

**Keywords:** Confucian values, relational-interdependent self, holistic thinking, self, thinking mode

## Abstract

As the primary value system in Chinese culture for almost 2,000 years, Confucianism has profoundly influenced the mindset of Chinese people. Cultural psychology studies have highlighted that individuals with different cultural backgrounds vary in their preferences for certain personality traits, such as self-construal, and their metacognitive characteristics, such as thinking modes. Compared with Western cultures, Chinese culture shows a preference for the interdependent self and holistic thinking. To investigate the relationship between the relational-interdependent self, holistic thinking, and traditional Chinese values (which are represented by Confucian values), we surveyed 327 Chinese adults using the Confucian Traditional Values Survey, Holistic Thinking Scale, and Relational-Interdependent Self-Construal Scale. The results show that Confucian values positively influence both holistic thinking and the relational-interdependent self, the latter of which partially mediates the positive relationship between Confucian values and holistic thinking. This study deepens the understanding of the psychological features of Chinese culture.

## Introduction

In psychological research, values are generally defined as “the ideological cores that drive personal decisions or actions” and “the principles and basic tenets that individuals tend to follow, and that guide their judgments regarding whether various behaviors are ‘good’ or ‘worthwhile,’ thus guiding and influencing individuals’ words and actions” (Zhu, 1990, pp. 93–97; Schwartz, 1997). In essence, values are individual or socio-cultural phenomena that are important predictors of the human mind and human behaviors (Klukhohn, 1951; Zhai, 1999).

From 134 BC—when Emperor Wu of the Han Dynasty adopted the policy of “forbidding all schools, venerating Confucianism only”—to the New Culture Movement of 1919 AD, a period of approximately 2,000 years, Confucianism was the dominant ideology in Chinese society and the core component of Chinese traditional values (Fung, 2011, p. 228). The Confucian value system is just as momentous to East Asian culture and East Asian society as is the ancient Greek value system to Western culture and Western society. The value preferences exhibited by contemporary Chinese people, and other East Asians, emphasize familism and clannishness, unity and harmony, self-discipline and self-moderation, filial piety, modesty and prudence, and respect for education, all of which reflect the typical characteristics of Confucian values (Yang, 1991, pp. 15–17; Hyun, 2001).

In cultural studies, Confucian values were once considered a major impediment to the modernization of East Asian countries (Weber, 1951). However, with the economic emergence of the “Four Asian Tigers” after the Cold War, it became necessary to reexamine Confucian values in terms of their benefits for economic growth. This led Kahn (1979) to propose the “post-Confucian hypothesis,” which argued that the four cultural traits of family orientation, group orientation, class orientation, and interpersonal orientation encapsulated in Confucian values are important sources of economic development in modern East Asia. Today, globalization and modernization are thriving in East Asia, especially China. Confucian values continue to shape and influence many psychological traits that are unique to Chinese and East Asians (Yang, 1991; Huang, 2006).

As a representative expression of the supreme pursuit of Confucian values, “unity of heaven and humanity” embodies the Confucian mode of recognizing and comprehending the world, that is, a holistic view of humankind and nature (Tang, 2005; Wang et al., 2021). It also embodies the ideal realm of the Confucian self, that is, to link oneself with many others and with everybody and everything in the universe as a whole (Liu, 2011; Wang et al., 2019). “Unity of heaven and humanity” is a concept derived from the *I Ching* (*The Book of Changes*) and emphasized throughout the history of Confucianism, from Confucius and Mencius in the pre-Qin period to the modern Neo-Confucians (Tang, 2005; Fung, 2014). Therefore, from a psychological perspective, the following questions need to be answered: what is the relationship between holistic thinking and the relational-interdependent self-construal—both of which represent Chinese cultural psychological characteristics—and traditional Confucian values? And how do Confucian values influence holistic thinking and the interdependent self? This study aimed to explore these issues and examine whether Confucian values can positively predict the holistic thinking mode through the mediating role of the relational-interdependent self-construal.

### Holistic thinking mode and Confucian values

Cultural psychology studies have shown that individuals’ thinking ways vary significantly across cultures (Bochner, 1994; Talhelm et al., 2014). Thinking mode refers to how people think and perceive the world; the thinking mode is formed through societies’ long-term historical development and, consequently, reflects the psychological characteristics of different cultures (Hou et al., 2016). Nisbett et al. (2001) conducted a series of in-depth studies on the differences between Western and Eastern thinking patterns and found that East Asians and Westerners perceive the world in very different ways. Westerners are inclined to use an analytical thinking mode and attend to a focal object, analyze its attributes, and categorize it to determine the rules that govern its behavior. By contrast, East Asians are more likely to use a holistic thinking mode, attend to a broad perceptual and conceptual field, notice relationships and changes, and group objects based on family resemblance rather than category membership. Similarly, Ji et al. (2000) suggested that Chinese people, when negotiating problems, tend to adopt a holistic cognitive orientation, emphasizing relationships and connections between things and integrating the problem with its context. By contrast, Americans tend to approach problems analytically, emphasizing the properties of things in their own right and using methods such as categorization to separate a thing from its context.

Peng and Nisbett (1999) connected the different preferences regarding thinking mode with the origins of Western and Eastern epistemological traditions. They argued that Western thinking follows the principles of Aristotle’s formal logic, with the law of identity, law of non-contradiction, and law of excluded middle as the basic rules. The correctness of a proposition is an either–or matter, without an intermediary; the essence of formal logic laws is to synthesize linear, logical, and unidirectional thoughts. Conversely, Eastern thinking, specifically Chinese thinking, prefers to regard the world as a universe of change, in which everything is a contradictory unity comprising opposites. Furthermore, when viewing and addressing problems, Eastern thinking focuses on dialectics and integrity. Overall, the application of the holistic thinking mode follows the principles of change, contradiction, and neutralization (Peng and Nisbett, 1999; Nisbett et al., 2001; Hou, 2007).

The above principles embodied in Chinese holistic thinking are consistent with Confucian values, such as the doctrine of the mean (*zhong-yong*), the pursuit of harmony, and the ideal of unity of heaven and humanity. The contrariety, coexistence, mutual dependence, and unity of opposites embodied in Chinese thinking are summarized in Chinese cultural psychology as “yin–yang thinking.” “Yin” and “yang” denote two opposite elements or dynamics that combine to produce all things. According to yin–yang thinking, all phenomena have both positive and negative sides, and the contradictory movements of yin and yang constitute the inherent basis for the continuous change in everything (Yang, 2006; Wang, 2018). The main feature of yin–yang thinking stems from the yin–yang theory presented in *I Ching*, the leading book among the *Six Confucian Classics* (Yang, 2006; Fung, 2011). The interpretation of the world and universe based on yin–yang thinking is both the ontological and epistemological basis of Chinese traditional philosophy. Therefore, Confucian values are directly related to holistic thinking. From the historical evolution perspective, the large-scale proliferation of holistic thinking in China and its emergence as the dominant thinking mode are associated with the promotion of yin–yang theory and the officialization of Confucian values that occurred during the Western Han Dynasty as a result of the efforts of Dong Zhong-Shu (Wang, 2018). Considering the above, it is clear that the influence of Confucian values on holistic thinking requires empirical confirmation.

Regarding the relationship between values and thinking modes, Li (2014) suggested that they are “intrinsically related and linked with each other, but also have relatively independent and different expressions with their own emphases.” Confucian values are expressed in the ideal of “inner saintliness, outer kingliness,” represented by the “Three Plateaus and Eight Entries” captured by the *Great Learning*, and its underlying doctrine is the theory of the original goodness of human nature and the humanistic view of the integration of “heaven, monarchy, and the people.” Such values form the basis of the Confucian thinking mode and cause Confucian thinking to manifest the holistic characteristics of “the unity of heaven and humanity” and the doctrine of the mean (Fung, 2011). Specifically, “the unity of heaven and humanity” is the framework of Confucian values, while the doctrine of the mean is the inner content. The texts of *Great Learning* and *Doctrine of the Mean*, respectively, represent exemplary expressions of the basic values and thinking modes of Confucianism (Li, 2014).

However, previous studies addressing the relationship between Confucian values and thinking modes are mostly based on philosophical or cultural theories and methods (Li, 2014; Wang, 2018). Thus far, there is neither empirical evidence in psychological studies supporting the correlation between Confucian values and the holistic thinking mode, nor any investigation of the pathways and underlying mechanisms of this relationship.

### Relational-interdependent self-construal and Confucian values

Self-construal, as a psychological characteristic closely related to thinking mode, is another important theme in cultural psychology (Triandis, 1989; Zhu et al., 2007). Markus and Kitayama (1991) developed the concept of self-construal, which suggested that individuals perceive their selves within specific cultural frames of reference, viewing the self through their culture’s perception of self–other relationships, especially the degree to which the self is separate from and connected to others. Markus and Kitayama (1991) initially divided self-construal into (1) the independent self, which is a bounded, unitary, stable entity that emphasizes an individual’s separation from the social context, and (2) the interdependent self, which is defined by social relationships and emphasizes connection with the social context; they suggested that the former is common in Western cultures, while the latter is usually prevalent in the East Asian cultural context. However, in a revision of their theory, Markus and Kitayama (2010) proposed that both types of self-construal generally exist in each cultural context and adjust to specific situations. There is not only separation but also mutual production, organization, and promotion between the two types.

The tripartite model of self, developed by Brewer and Gardner (1996), further distinguished between the basic forms of self-representation that exist in different cultures. Brewer and Gardner (1996) argued that the self-concept comprises three fundamental self-representations: the individual self, the relational self, and the collective self. The individual self refers to the self-concept that distinguishes oneself from others and emphasizes oneself as a collection of certain unique traits and personalities and separates from the social context in which one lives. The relational self is defined by one’s relationships with significant others and is based on interpersonal relations, such as parent–child, friend, romantic partner, and other specific role relationships (e.g., teacher–student and doctor–patient). Finally, the collective self is defined as the inclusion of oneself in certain types of social groups and contains self-concepts that distinguish in-group members from out-group members, often comparing the group to which one belongs (in-group) with other groups (out-groups; Sedikides and Brewer, 2015).

Synthesizing the interdependent self of self-construal theory and the relational self of the tripartite model of self, Cross et al. (2000) proposed the relational-interdependent self-construal (RISC) as a means of defining the self based on one’s intimate relationships with others. The RISC emphasizes paired interpersonal relationships (e.g., mother–child relationships) and the inclusion of significant others (e.g., parents, spouse, friends) and their relationships in one’s self-concept, which is achieved by actively building and consolidating good relationships with others (Huang and Bi, 2012; Jiang et al., 2017).

According to Cross et al. (2011), self-construal reflects one’s self-view within a certain culture. Social customs and institutions based on cultural values implicitly permeate people’s thinking modes, language use, educational systems, and even management systems; this provides a context for their behaviors and creates general preferences regarding the presentation of the self (Boucher, 2014). From the perspective of cultural psychology, the relational-interdependent self corresponds more to the self-presentation promoted by traditional East Asian cultures than in Western cultures. Eastern and Western self-presentation generally differ in the aspect of the perceived relationship between the self and others. The Western self emphasizes individual subjectivity, independence, and separation from others, while the Chinese self is based on “human relations” and focuses on embedding the self in a network of social relationships (Zhai, 2018; Wang et al., 2019).

Emphasis on interpersonal relationships is an important aspect of Confucian values. The core concepts of Confucianism, such as “benevolence,” “propriety,” “loyalty,” and “forgiveness,” are all constructed around human relationships (Li, 2014). In terms of historical evolution, Wang (2019) demonstrated that Confucian values have influenced the emergence and development of the relational-interdependent characteristics of the Chinese self. Philosophical and sociological studies about the Confucian self almost always emphasize ethical roles and relationships rather than a unique individual (see Ho, 1995; Liang, 2005; Fei, 2008; Yang, 2009; Ames, 2017; Zhai, 2018). Wang (2021) summarized the main characteristics of the self advocated by Confucian values as (1) interdependence, in which the boundaries between the other and the self are blurred, flexible, and elastic and the self is embedded in a more macroscopic social network, and (2) relationism, which is typified by the self-presentation that Confucian values promote and emphasizes the ethical positioning of roles, such that when referring to someone in traditional vernacular society, one often does not call them by their name, but rather by “what relationships they are of whom,” with the possessive case generally being those who are more closely related to the speaker. The “relational-interdependent self” studied in cultural psychology is closely related to the self-presentation promoted by Confucian values. This begs the following question: Does the effect of Confucian values on the relational interdependence of the self influence the holistic thinking mode promoted by Confucianism? This study aimed to answer this question.

### The present study

Several studies have compared Westerners and East Asians in terms of similarities and differences in self-construal and thinking modes. The results have demonstrated that self-construal and thinking modes are often closely related within a given culture (see Kitayama and Markus, 1999; Yang, 2006; Spencer-Rodgers et al., 2009; Boucher, 2011; Talhelm et al., 2014). Although there is no empirical research that directly explores the underlying mechanisms of the effect of the self on thinking, the notion that thinking mode is influenced by the self is implicit in many existing cultural psychology theories. Moore (1968) and Hansen (1983) argued that Chinese people see the world as a whole of intertwined things in that they emphasize interpersonal relationships; thus, they constantly try to understand things on the basis of this complexity, and their analysis of things is not limited to the things themselves, but often includes contexts and environments. Western values originate from the ancient Greek civilization, which believed that the world consists of countless individual properties that can be regarded as independent objects, and each object has its own characteristics that can be separated from the whole (Wang et al., 2021). Therefore, Westerners are more proficient at analyzing the characteristics of objects and, thus, developing a clearer understanding of the essences of things (Hou et al., 2016). Nisbett and Masuda (2003) took the example of different cultural patterns regarding infant rearing to demonstrate that, when compared with other cultures, Asian infants develop stronger ties to their caregivers and have more complex relationships in the social environments in which they grow up, rendering them better able to incorporate significant others into their self-construal. This influences their cognitive traits, leading them to have a propensity for focusing on scenarios as a whole rather than as separate objects. The opposite is true for people raised in European and American cultures, where the simpler and fewer the social relationships, the more likely they are to view the attributes of primary objects in isolation from their surroundings, focus more on the interpretation of a single object, view the world as discrete and discontinuous, and have a greater sense of control over themselves (Nisbett et al., 2001). In sum, the emphasis on interdependence or independence of self-construal varies across cultures, thereby leading to differences in the metacognitive characteristics of the respective thinking modes.

**FIGURE 1:**
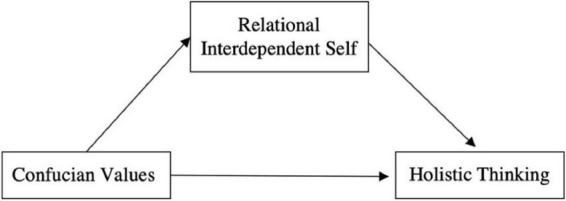
Hypothetical model for Confucian values, relational-interdependent self, and holistic thinking.

This study aimed to explore the relationship between Confucian values, RISC, and the holistic thinking mode. Based on the above review, we hypothesized that the more individuals identify with Confucian values, the more they tend to have a relational-interdependent self, which, in turn, leads to a preference for holistic thinking; that is, their thinking conforms to the principles of change, contradiction, and relationism or holism. The hypothetical model for this relationship is illustrated in Figure 1.

## Materials and methods

### Participants

The questionnaire used for this research was created on the Questionnaire Star website,^1^ and for participant recruitment, the link to the questionnaire was sent to WeChat groups. Each participant voluntarily agreed to complete the survey and signed an informed consent form. Interspersed among the main questionnaire items were four detection questions designed to test the participants’ authenticity and earnest. Our inclusion criteria were as follows: (1) age ≥ 18 years, (2) Chinese-speaking, and (3) resident on the Chinese mainland. Specifically, we excluded participants who (1) completed the survey in < 300 s, which is the minimum time required to read through the demographic questionnaire and the three scales (*N* = 9), and (2) provided wrong responses to any of the detection questions (*N* = 42, of which six also did not meet the time requirement). A total of 402 questionnaires were distributed, of which 45 respondents were excluded according to the above standards, thus resulting in a final sample of 357 participants whose data were included in the current analysis. The mean age of the valid participants was 25.26 years (SD = 6.80). Among them, 249 were female and 108 were male; overall, 63 had an education level of a high school degree or lower, 191 had a bachelor’s degree, and 103 had a master’s degree. Most of the valid participants were from Jiangsu (14.85%), Shandong (13.45%), Hebei (6.72%), or Guangdong (5.04%).

### Measures

#### Confucian traditional values survey

To measure the participants’ level of Confucian values, we used the Confucian Traditional Values Survey, which was developed by Yang (2004) with reference to Bond’s “Chinese Values Scale,” Kahn’s Post-Confucian hypothesis, and Lau’s conception of Chinese materialism. The scale contains 40 items, each of which is rated using a four-point Likert scale ranging from “not important at all” to “extremely important.” The respondents use the scale to indicate the degree of importance they place on the topic of focus for each item. Higher total scores indicate a greater tendency to identify with traditional Confucian values. The scale contains five factors: familism, modesty and humility, face-saving relationships, unity and harmony, and tenacity and diligence. At the time of the scale’s initial development, the internal consistency coefficients for the five factors were 0.87, 0.82, 0.71, 0.84, and 0.6, respectively (Yang, 2004). For the current study, the Cronbach’s α for the entire scale was 0.928, while for the five factors, the values were 0.869, 0.808, 0.785, 0.857, and 0.664, respectively. Confirmatory factor analysis (CFA) of the scale showed that Chi-square/degrees of freedom (χ^2^*/df*) = 2.661, root mean square error of approximation (RMSEA) = 0.068, Tucker–Lewis index (TLI) = 0.983, confirmatory fit index (CFI) = 0.993, and standardized root mean square residual (SRMR) = 0.021.

#### Holistic thinking scale

To measure the participants’ holistic thinking, the Holistic Thinking Scale developed by Hou et al. (2016), which was created by referring to Peng and Nisbett’s (2000) Chinese Dialectical Thinking Scale, and Chiu’s (2000) Chinese Zhong-Yong Thinking Scale, was adopted in the current study. This scale comprises 13 items, each of which is scored using a seven-point Likert scale ranging from 1 (“strongly disagree”) to 7 (“strongly agree”); the items measure one of three dimensions: connectivity, change, and contradiction. Higher total scores indicate higher levels of holistic thinking. In its original development, the scale showed good model fit indices (χ^2^/*df* = 1.386, RMSEA = 0.044, GFI = 0.940, CFI = 0.870); internal consistencies of 0.761, 0.62, and 0.61 for the connectivity, change, and contradiction dimensions, respectively; and retest reliability values of 0.79, 0.67, and 0.72, respectively. For the current study, the Cronbach’s α for the scale was 0.783, and the fit indices for the CFA were χ^2^/*df* = 2.551, RMSEA = 0.066, TLI = 0.825, CFI = 0.865, and SRMR = 0.076.

#### Relational-interdependent self-construal scale

The Chinese version of the Relational-Interdependent Self-Construal Scale (Huang et al., 2012), originally developed by Cross et al. (2000) to further differentiate between the types of interdependent self, was used in the current study. The Chinese version, which is similar to the original, uses a seven-point Likert scale ranging from 1 (“strongly disagree”) to 7 (“strongly agree”) and contains nine items (the original English language version contains 11 items; in the Chinese revision, items 8 and 9 from the English version were weakly homogeneous with the total score and were therefore deleted). This scale contains only one dimension: the relational-interdependent self. Higher total scores indicate stronger relational interdependence regarding self-construal. The Chinese version of this scale showed good model fit indices (χ^2^/*df* = 1.280, RMSEA = 0.030, NFI = 0.890, CFI = 0.870), and the internal consistency reliability was 0.73. For the current study, the Cronbach’s α was 0.823, and the fit indices for the CFA were χ^2^/*df* = 3.009, RMSEA = 0.075, TLI = 0.915, CFI = 0.936, and SRMR = 0.047.

### Statistical analysis

IBM SPSS Statistics 26.0 was used to analyze the demographic variables and to conduct Pearson’s product-moment correlation analysis to examine the correlations between Confucian values, holistic thinking, and relational-interdependent self. Mplus 8.3 was used to develop structural equation models, with the maximum likelihood method being used to test the significance of the mediating role of the relational-interdependent self in the relationship between Confucian values and holistic thinking.

### Common method bias control

As all three measurement tools used in the current study were self-report scales, there was a risk of common method bias; however, the following elements of the research design and the scales themselves minimized this risk: (1) all questionnaires were anonymous; (2) items were scored using four-point or seven-point Likert-type scales, which could inhibit the participants’ habitual responses, and the order of the three questionnaires was counterbalanced across the participants using the Latin square design; (3) the scales adopted in the current study had high reliability and validity and thus minimized or avoided systematic errors in measurement; (4) some items and dimensions in the questionnaire were reverse-scored; and (5) the participants were recruited from various geographical areas, which increased the variation in the dataset. In addition, after data collection, common method bias was further tested using Harman’s one-way test. A total of 15 factors showed eigenvalues greater than 1 in the unrotated case, and the variance ratio explained by the first factor was 21.444%, which was less than the critical criterion of 40%; this indicated that the study did not have a serious common method bias problem (Zhou and Long, 2004).

## Results

The variables did not differ significantly in terms of the participants’ gender, age, educational level, or geographical location; therefore, these variables did not need to be controlled for in following analysis.

### Correlation between Confucian values, holistic thinking, and relational-interdependent self

As shown in Table 1, there was a significant positive correlation between Confucian values, the relational-interdependent self, and holistic thinking (*r* = 0.41–0.55, *p* < 0.001), and the dimensions of the variables showed different degrees of significant positive correlation (*r* = 0.14–0.88, *p* < 0.01). The results of the correlation analysis were consistent with the hypothesis and could be further tested.

**TABLE 1:** Correlation matrix for Confucian values, holistic thinking, and relational-interdependent self.

	1	2	3	4	5	6	7	8	9	10
1. Confucian values	–									
2. Familism	0.88**	–								
3. Modesty and humility	0.88**	0.65**	–							
4. Face-saving relationships	0.82**	0.54**	0.73**	–						
5. Unity and harmony	0.86**	0.85**	0.66**	0.55**	–					
6. Tenacity and diligence	0.67**	0.53**	0.63**	0.50**	0.50**	–				
7. Holistic thinking	0.41**	0.30**	0.40**	0.40**	0.30**	0.28**	–			
8. Connectivity	0.34**	0.28**	0.31**	0.26**	0.32**	0.22**	0.76**	–		
9. Change	0.32**	0.24**	0.32**	0.27**	0.24**	0.22**	0.77**	0.44**	–	
10. Contradiction	0.26**	0.17**	0.28**	0.27**	0.14**	0.19**	0.72**	0.36**	0.23**	–
11. Relational-interdependent self	0.46**	0.38**	0.40**	0.35**	0.40**	0.26**	0.55**	0.46**	0.42**	0.36**

*p < 0.05; **p < 0.01.

### Model fit test

Based on the correlation analysis results, a structural equation model with Confucian values as the independent variable, holistic thinking as the dependent variable, and relational-interdependent self as the mediator was constructed. The model fit indices were all within the acceptable range (see Table 2): χ^2^/*df* < 5, RMSEA < 0.08, TLI and CFI > 0.9, and SRMR < 0.05.

**TABLE 2:** Model fit index.

	χ^2^	*df*	χ^2^/*df*	RMSEA [90% CI]	TLI	CFI	SRMR
Model	44.204	24	1.841	0.049 [0.025, 0.071]	0.980	0.986	0.028

CFI, confirmatory fit index; CI, confidence interval; df, degrees of freedom; TLI, Tucker–Lewis index; RMSEA, root mean square error of approximation; SRMR, standardized root mean square residual.

### Mediating effect test

**FIGURE 2:**
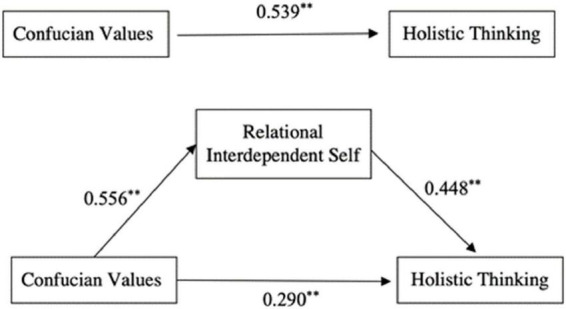
Structural model of the mediating effect of the relational-interdependent self on the relationship between Confucian values and holistic thinking. *p < 0.05; **p < 0.01.

The test of joint significance (Hayes, 2013) showed that the overall mediating effect was significant. A bias-corrected non-parametric percentage bootstrap test was used with 2,000 replicate samples, and 95% confidence intervals (CIs) were calculated. The results of the structural model for the mediation analysis are presented in Figure 2; the results show that the coefficients for each path in the model were highly significant (*p* < 0.001).

**FIGURE 3:**
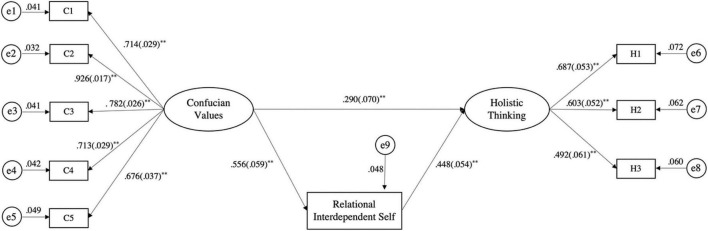
Latent variable path of the linear equation model. *p < 0.05; **p < 0.01. C1–C5 represent the five factors of Confucian values (C1, familism; C2, modesty and humility; C3, face-saving relationships; C4, unity and harmony; C5, tenacity and diligence); H1–H3 represent the three dimensions of holistic thinking (H1, connectivity; H2, change; H3, contradiction).

As shown in Figure 3, the five dimensions of Confucian values (i.e., familism, modesty and humility, face-saving relationships, unity and harmony, and tenacity and diligence) as well as the three dimensions of holistic thinking (connectivity, change, and contradiction) had significant effects in this model (*p* < 0.001).

As shown in Table 3, the direct effect of Confucian values on holistic thinking in the model path was 0.290, accounting for 53.80% of the total effect, and the indirect effect was 0.249, accounting for 46.2% of the total effect. The 99% CI corresponding to the indirect path did not contain 0, verifying the mediating role of the relational-interdependent self in the relationship between Confucian values and holistic thinking.

**TABLE 3:** Path effects analysis.

Mediating effects paths	Estimated value	SE	99% CI^a^
Total effect (CV→RISC→HT)	0.539	0.067	[0.358, 0.699]
Indirect effect (CV→RISC→HT)	0.249	0.040	[0.159, 0.375]
Direct effect (CV→HT)	0.290	0.069	[0.112, 0.546]

^a^The difference is significant if the confidence interval does not contain 0, and vice versa. CI, confidence interval; SE, standard error.

In sum, the relationship between Confucian values, relational-interdependent self, and holistic thinking supports the above model.

## Discussion

The current study explored the relationship between Confucian values, RISC, and holistic thinking modes and verified that there was a significant two-way positive correlation between them. This indicates that the more people hold Confucian values or believe in Confucianism, the stronger the relational interdependence of their self-construal and the more holistic their thinking. Moreover, the mediating effect of the relational-interdependent self on the relationship between Confucian values and holistic thinking was demonstrated; that is, the direct effect of Confucian values on holistic thinking and its indirect effect through the mediation of relational-interdependent self were verified.

First, our hypothesis that Confucian values influence holistic thinking was verified. According to previous studies in cultural psychology, holistic thinking is one of the most typical Chinese psychological characteristics (Talhelm et al., 2014; Wang, 2018). Chinese people’s holistic thinking mode is related to the Confucian epistemology of seeing and understanding the world, which emphasizes that each individual is embedded in a large and complex network of relationships and contexts (Yang, 2009). Particularly, Confucianism emphasizes the “unity of heaven and humanity” and the merging of man and nature; this opposes the fragmentation of the relationship between human and nature and, as stated in *Wen Yan*, *I Ching*, promotes the “harmony with heaven and earth, the harmony with the sun and moon, and the harmony with the order of the four seasons.” This view has a direct influence on the holistic thinking of Chinese people (Fung, 2014). Moreover, the doctrine of the mean (*zhong-yong*) promoted by Confucianism stresses that things should be viewed with a dynamic, developmental, and balanced perspective, grasping the “middle” while also seeing both contradictory sides (Chiu, 2000). Some Chinese psychologists summarize Confucian thinking characteristics as zhong-yong thinking, which refers to “considering things carefully from different aspects and conducting appropriate behaviors to account for entire situations” (Yang, 2010), which is the embodiment of the holistic thinking mode in the philosophy of Chinese life practice (Chiu, 2000; Wu and Lin, 2005; Gao, 2021). In recent cross-cultural comparative studies, Chinese participants have been found to place greater emphasis on tolerating contradictions, anticipating change, and viewing problems holistically when compared with Western participants (Nisbett et al., 2001; Talhelm et al., 2014; Wang, 2018). The current study builds on such results and reveals the deeper mechanism underlying them, that is, the influence that Confucian values have on the holistic thinking mode.

Additionally, our findings concerning Confucian values’ influence on the relational-interdependent self were consistent with our hypothetical expectations. Zhai (2018) summarized the theoretical research on the Confucian self discussed in Neo-Confucianism and sociology in the twentieth century, pointing out that the Confucian self, which describes the relational-interdependent self formally influenced by Confucian values, is the base of the Chinese self. Cross et al. (2000) suggested that relational interdependence is fundamentally different from collective interdependence. The latter type places greater emphasis on the subordination of individuals within a group; exemplars are the Chinese Mohists or certain religious groups in the West, whose values tend to promote a kind of “universal love” or “philanthropism” that emphasizes equality. By contrast, Confucian values advocate a gradual and progressive benevolence from cultivating oneself, family harmony, moving toward country management, and eventually world peace and emphasize differentials in order (Wang and Wang, 2021). In short, Confucian values advocate a kind of “self-extension,” meaning “extending oneself to others,” which refers to accommodating self-development (in a pro-social sense) rather than conquering expansion (Yang, 2006; Wang et al., 2019). The supreme goal of self-extension is achieving “unity of heaven and humanity,” in which the self will accommodate everyone and even everything (Tang, 2005; Liu, 2011). Therefore, it is inaccurate, in traditional cultural studies, to categorize Chinese and East Asian cultures as simply “collectivism.”

The current study also provides an alternative interpretation of the relationship between Confucian values and holistic thinking by developing a structural equation model, which demonstrated that the relational-interdependent self serves as a mediator between them. Specifically, Confucian values can influence holistic thinking directly and indirectly through the relational-interdependent self. Spencer-Rodgers et al. (2009) ascertained that one’s self-conceptions are indicative of folk epistemologies and thinking modes. Self-conceptions and self-construal trigger people to think about their life experiences and social world. As Markus and Kitayama (1991) argued, if people see themselves as dependent beings embedded in a broader context, they are likely to perceive surrounding objects or events in a similar way. That is, people with interdependent selves are better able to naturally relate all things to their contexts because they can place themselves within a larger whole and promote harmony between themselves and their surroundings. This may be related to long-standing forms of social organization. The ancient Greek city-state system focused on individuals’ contractual spirit in public life and emphasized the rights and obligations of each individual as a separate entity, thereby inculcating the logic system (Fung, 1922; Peng and Nisbett, 1999). By contrast, the patriarchal clan system that has existed in China since the Western Zhou dynasty emphasizes relational connections between individuals, using blood and blood-like ties to bind one’s self to the identity traits defined by his/her social relationships. Confucian values advocate a ritual and music system based on the patriarchal clan and enfeoffment systems, thereby further promoting self-construal toward the direction of relational interdependence (Fung, 2011). Individuals with a Confucian cultural background imbued with such relational ties tend to think in more complex networks and in larger contexts.

Although previous studies have addressed the relationship between cultural values and self-construal, cultural values and thinking modes, or thinking values and self-construal from either theoretical or empirical perspectives (e.g., Markus and Kitayama, 1991, 2010; Nisbett and Masuda, 2003; Yang, 2004; Wang, 2018, 2019), few empirical studies have been conducted to explore the relationship between all three concepts. This study, although a preliminary attempt, fills this gap in the literature and lays the foundation for further causality studies.

In previous studies, holistic thinking and the interdependent self have been identified as signature psychological traits of East Asians (Peng and Nisbett, 1999; Yang and Lu, 2009; Zhu and Ng, 2017). The results of this study—which proves that self-construal and thinking modes are not culturally isolated or static psychological traits but instead are significantly influenced by socio-cultural factors—provide a way to understand the personality as well as the thinking patterns of East Asians. Furthermore, the significance of studying Confucian values is not limited to the context of East Asian cultures; indeed, they are a presentation of psychological patterns that influence human personality and thinking in a socio-cultural context. These finds are also the response to the advocacy of “one mind, many mentalities” in cultural psychology (Shweder et al., 2007; Henrich et al., 2010).

In summary, the present work explored the interrelationship between Confucian values, the relational-interdependent self, and holistic thinking in the Chinese cultural context. The present findings regarding the partial mediating role of the relational-interdependent self provide a basis for further exploration of the formation mechanism of Chinese holistic thinking. However, this study has some limitations worth noting. First, although our sample size was sufficient to validate the hypothesis model, it was insufficient to explore the role of geographical, age, and intergenerational effects. In future research, it would be worth employing cross-cultural or cross-subcultural samples, as well as examining a wider range of ages. Besides, the structural equation model only verified the quasi-causal relationships in the hypotheses, which were derived from existing theories (Wen and Ye, 2014). However, we acknowledge that the correlational nature of our design does not provide strong evidence for causal mediation and prevents us from concluding whether there is a better model (Kline, 2015). A design with an experimental approach would provide evidence for causal mediation (Spencer et al., 2005). Therefore, in future research, the causal relationships between cultural values, self-construal, and thinking modes should be explored more effectively, such as by using initiation or intervention methods to manipulate cultural value variables to examine their direct effects on self-construal and thinking modes. Third, this study focuses only on the effect of Confucian values on the cultural and psychological characteristics of Chinese people. Although Confucianism was the most profound ideological system in ancient China, the traditional Chinese culture is regarded as a trinity of Confucianism, Taoism, and Buddhism (Fung, 2011; Wang and Wang, 2021); it is worthwhile to explore the effect of Taoist values and Buddhist values on the Chinese thinking and self. Finally, at the sociological level, China has, in the last century, experienced unprecedented value and social structural transition as a result of Westernization, globalization, and a series of political movements; consequently, traditional Chinese value systems face collapse and disintegration (Lu, 2013; Cai et al., 2020). It would be worthwhile for cultural scholars to investigate how changes in values will lead to changes in the self-construal and thinking mode of Chinese people from a longer historical vicissitude perspective.

## Data availability statement

The raw data supporting the conclusions of this article will be made available by the authors, without undue reservation.

## Ethics statement

The studies involving human participants were reviewed and approved by the Ethics Committee of Nanjing Normal University. The patients/participants provided their written informed consent to participate in this study.

## Author contributions

Z-DW contributed to the conception and design of the study, data collection and discussion, and drafting the article. Y-MW and HG contributed to data collection and data analysis and discussion. QZ contributed to the critical revision of the manuscript and final approval of the version to be published. All authors contributed to the article and approved the submitted version.

## Footnotes

1
www.wjx.cn

## References

[B1] Ames R. T. (2017). *Confucian roles ethics: A vocabulary.* Jinan: Shandong People’s Publishing House.

[B2] Bochner S. (1994). Cross-cultural differences in the self concept: A test of Hofstede’s individualism/collectivism distinction. *J. Cross Cult. Psychol.* 25 273–283. 10.1177/0022022194252007

[B3] Boucher H. C. (2011). The dialectical self-concept II: Cross-role and within-role consistency, well-being, self-certainty, and authenticity. *J. Cross Cult. Psychol.* 42 1251–1271. 10.1177/0022022110383316

[B4] Boucher H. C. (2014). The relational-interdependent self-construal and positive illusions in friendship. *Self Identity* 13 460–476. 10.1080/15298868.2013.843472

[B5] Brewer M. B. Gardner W. (1996). Who is this “We”? Levels of collective identity and self representations. *J. Pers. Soc. Psychol.* 71 83–93. 10.1037/0022-3514.71.1.83

[B6] Cai H. J. Huang Z. H. Lin L. Zhang M. Y. Wang X. O. Zhu H. J. (2020). The psychological change of the Chinese people over the past half century: A literature review. *Adv. Psychol. Sci.* 28 2068–2080. 10.3724/SP.J.1042.2020.01599

[B7] Chiu C.-Y. (2000). Assessment of Zhong-Yong (dialectic) thinking: Preliminary findings from a cross-regional study. *Hong Kong J. Soc. Sci.* 18 33–54.

[B8] Cross S. E. Bacon P. L. Morris M. L. (2000). The relational-interdependent self-construal and relationships. *J. Pers. Soc. Psychol.* 78 791–808. 10.1037/0022-3514.78.4.791 10794381

[B9] Cross S. E. Hardin E. E. Gercekswing B. (2011). The what, how, why, and where of self-construal. *Pers. Soc. Psychol. Rev.* 15 142–179. 10.1177/1088868310373752 20716643

[B10] Fei H. T. (2008). *From the soil: The foundations of Chinese society.* Beijing: People’s Publishing House.

[B11] Fung Y. L. (1922). Why China has no science - an interpretation of the history and consequences of Chinese philosophy. *Int. J. Ethics* 32 237–263. 10.1086/intejethi.32.3.2377487

[B12] Fung Y. L. (2011). *History of Chinese philosophy.* Shanghai: East China Normal University Press.

[B13] Fung Y. L. (2014). *Six books of Zhen Yuan.* Beijing: Zhonghua Book Company.

[B14] Gao Z. Q. (2021). The cultural psychological characteristics of the golden mean and its theoretical path of practice. *J. Psychol. Sci.* 44 1018–1023.

[B15] Hansen C. (1983). *Language and logic in ancient China.* Ann Arbor: University of Michigan Press.

[B16] Hayes A. F. (2013). *Introduction to mediation, moderation, and conditional process analysis: A regression-based approach.* New York, NY: Guilford Press.

[B17] Henrich J. Heine S. J. Norenzayan A. (2010). Beyond WEIRD: Towards a broad-based behavioral science. *Behav. Brain Sci.* 33 111–135. 10.1017/S0140525X10000725

[B18] Ho D. Y. F. (1995). Selfhood and identity in confucianism, taoism, buddhism, and hinduism: Contrasts with the west. *J. Theory Soc. Behav.* 25 115–139. 10.1111/j.1468-5914.1995.tb00269.x

[B19] Hou Y. B. (2007). Research progress in thinking styles from the perspective of cultural psychology. *Adv. Psychol. Sci.* 15 211–216.

[B20] Hou Y. B. Peng K. P. Zhu Y. (2016). Chinese thinking styles: Their concept and structure. *Chin. Soc. Psychol. Rev.* 11 45–72. 32936505

[B21] Huang L. Bi Z. Z. (2012). The reliability and validity of the Chinese version of the relational-interdependent self-construal scale. *Adv. Psychol.* 2 173–178. 10.12677/AP.2012.24027

[B22] Huang K. K. (2006). *Confucian relationism: Cultural reflection and paradigm reconstruction.* Beijng: Peking University Press.

[B23] Hyun K. J. (2001). Sociocultural change and traditional values: Confucian values among Koreans and Korean Americans. *Int. J. Intercult. Relat.* 25 203–229. 10.1016/S0147-1767(01)00009-8

[B24] Ji L. J. Peng K. P. Nisbett R. E. (2000). Culture, control, and perception of the environment. *J. Pers. Soc. Psychol.* 78 943–955. 10.1037/0022-3514.78.5.943 10821200

[B25] Jiang T. Canevello A. Gore J. S. Hahn J. H. Crocker J. (2017). The association between compassionate goals and relational-interdependent self-construal. *Self Identity* 16 143–170. 10.1080/15298868.2016.1238406 29200979 PMC5704777

[B26] Kahn H. (1979). *World economy development, 1979 and beyond.* London: Croom Helm.

[B27] Kitayama S. Markus H. R. (1999). “Yin and yang of the japanese self: The cultural psychology of personality coherence,” in *The coherence of personality: Social-cognitive bases of consistency, variability, and organization*, eds Cervone D. Shoda Y. (New York, NY: Guilford Press), 242–302.

[B28] Kline R. B. (2015). *Principles and practice of structural equation modeling.* New York, NY: Guilford publications.

[B29] Klukhohn C. K. M. (1951). “Values and value orientations in the theory of action,” in *Toward a general theory of action*, eds Parsons T. E. Shils E. A. (Cambridge, MA: Harvard University Press), 388–433. 10.4159/harvard.9780674863507.c8

[B30] Li M. (2014). An introduction to the basic values and ways of thinking of confucianism. *Confucian Stud.* 6 101–106.

[B31] Liang S. M. (2005). *Essence of Chinese culture.* Shanghai: Shanghai People’s Publishing House.

[B32] Liu X. G. (2011). On the identity and positioning of Chinese philosophical studies: A case study of Tian-ren-he-yi (Unity of Heaven and Man) in ancient and modern times. *J. Nanjing Univ.* 48 67–156.

[B33] Lu L. (2013). Value reorganization in a changing society. *Appl. Psychol. Res.* 58 11–184.

[B34] Markus H. R. Kitayama S. (1991). Culture and the self: Implications for cognition, emotion, and motivation. *Psychol. Rev.* 98 223–253. 10.1037/0033-295X.98.2.224

[B35] Markus H. R. Kitayama S. (2010). Cultures and selves: A cycle of mutual constitution. *Perspect. Psychol. Sci.* 5 420–430. 10.1177/1745691610375557 26162188

[B36] Moore C. A. (1968). *The Chinese mind: Essentials of Chinese philosophy and culture.* Honolulu: University of Hawaii Press. 10.1515/9780824844912

[B37] Nisbett R. E. Masuda T. (2003). Culture and point of view. *Proc. Natl. Acad. Sci. U.S.A.* 100 11163–11170. 10.1073/pnas.1934527100 12960375 PMC196945

[B38] Nisbett R. E. Peng K. Choi I. Norenzayan A. (2001). Culture and systems of thought: Holistic versus analytic cognition. *Psychol. Rev.* 108 291–310. 10.1037/0033-295X.108.2.291 11381831

[B39] Peng K. Nisbett R. E. (1999). Culture, dialectics, and reasoning about contradiction. *Am. Psychol.* 54 741–754. 10.1037/0003-066X.54.9.741

[B40] Peng K. Nisbett R. E. (2000). Dialectical responses to questions about dialectical thinking. *Am. Psychol.* 55 1067–1075. 10.1037//0003-066X.55.9.1067 11036717

[B41] Schwartz S. H. (1997). *Values and culture.* London: Routledge.

[B42] Sedikides C. Brewer M. B. (2015). *Individual self, relational self, collective self.* New York, NY: Psychology Press. 10.4324/9781315783024

[B43] Shweder R. A. Goodnow J. Hatano G. Levine R. Markus H. Miller P. (2007). “The cultural psychology of development: One mind, many mentalities,” in *Handbook of child psychology*, ed. Damon W. (New York, NY: Wiley), 716–792. 10.1002/9780470147658.chpsy0113

[B44] Spencer-Rodgers J. Boucher H. C. Morri S. C. Lei W. Peng K. (2009). The dialectical self-concept: Contradiction, change, and holism in east asian cultures. *Pers. Soc. Psychol. Bull.* 35:29. 10.1177/0146167208325772 19106076 PMC2811254

[B45] Spencer S. J. Zanna M. P. Fong G. T. (2005). Establishing a causal chain: Why experiments are often more effective than mediational analyses in examining psychological processes. *J. Pers. Soc. Psychol.* 89 845–851. 10.1037/0022-3514.89.6.845 16393019

[B46] Talhelm T. Zhang X. Oishi S. Shimin C. Duan D. Lan X. (2014). Large-scale psychological differences within China explained by rice versus wheat agriculture. *Science* 344 603–608. 10.1126/science.1246850 24812395

[B47] Tang Y. J. (2005). Discussion on the “unity of heaven and human.” *Hist. Chin. Philos.* 2 5–10.

[B48] Triandis H. C. (1989). The self and social behavior in differing cultural contexts. *Psychol. Rev.* 96 506–520. 10.1037/0033-295X.96.3.506

[B49] Wang F. Y. (2018). Questioning the rice theory: Also on the internal and external causes of Chinese preference for holistic thinking. *Acta Psychol. Sin.* 50 572–582. 10.3724/SP.J.1041.2018.00572

[B50] Wang F. Y. (2019). Independent self and interdependent self: Their emergence, transformation and the formation of Chinese self-construal viewed from the perspective of the evolution of culture and history. *J. Nanjing Normal Univ.* 4 61–77.

[B51] Wang F. Y. Wang Z. D. Wang R. J. (2019). The Taiji model of self. *Front. Psychol.* 10:1443. 10.3389/fpsyg.2019.01443 31293484 PMC6598445

[B52] Wang Z. D. (2021). *Differential altruism of Chinese people: The effect and mechanism of cultural self-construal.* Doctoral dissertation. Nanjing: Nanjing Normal University.

[B53] Wang Z. D. Wang F. Y. (2021). Ternary Taiji models of the traditional Chinese self: Centered on confucian, taoist, and buddhist cultures. *J. Humanist. Psychol.* 10.1177/00221678211016957

[B54] Wang Z. D. Wang Y. M. Li K. Shi J. Wang F. Y. (2021). The comparison of the wisdom view in Chinese and Western cultures. *Curr. Psychol.* 10.1007/s12144-020-01226-w 33424207 PMC7786156

[B55] Weber M. (1951). *The religion of China: Confucianism and taoism.* New York, NY: Free Press.

[B56] Wen Z. L. Ye B. J. (2014). Analyses of mediating effects: The development of methods and models. *Adv. Psychol. Sci.* 22 731–745. 10.3724/SP.J.1042.2014.00731

[B57] Wu C. H. Lin Y. C. (2005). Development of a Zhong-Yong thinking style scale. *Indig. Psychol. Chin. Soc.* 24 247–300.

[B58] Yang C. F. (1991). *Chinese people and Chinese mind.* Taipei: Yuan-Liou Publishing Company.

[B59] Yang C. F. (2006). “The Chinese conception of the self,” in *Indigenous and cultural psychology*, eds Kim U. Yang K. S. Hwang K. K. (Boston, MA: Springer), 327–356. 10.1007/0-387-28662-4_15

[B60] Yang C. F. (2010). Multiplicity of Zhong Yong studies. *Indig. Psychol. Chin. Soc.* 34 3–39.

[B61] Yang K. S. (2004). *The psychology and behavior of Chinese people.* Beijing: China Renmin University Press.

[B62] Yang K. S. Lu L. (2009). *Chinese self: Analysis with psychology.* Chongqing: Chongqing University Press.

[B63] Yang Y. Y. (2009). Guanxilization or categorization: Psychological mechanisms contributing to the formation of the Chinese concept of “us”. *Soc. Sci. China* 4 49–67. 10.1080/02529200902903800

[B64] Zhai X. W. (1999). Chinese values: Types, transformations and their problems. *J. Nanjing Univ.* 4 118–126.

[B65] Zhai X. W. (2018). Confucian-style self and its practice: A study of indigenous psychology. *Nankai J.* 5 129–139.

[B66] Zhou H. Long L. R. (2004). Statistical remedies for common method biases. *Adv. Psychol. Sci.* 12 942–950.

[B67] Zhu Y. Ng S. H. (2017). *Looking for the Chinese self.* Beijing: Beijing Normal University Press.

[B68] Zhu Y. Zhang L. Fan J. Han S. (2007). Neural basis of cultural influence on self-representation. *Neuroimage* 34 1310–1316. 17134915 10.1016/j.neuroimage.2006.08.047

[B69] Zhu Z. X. (1990). *The comprehensive dictionary of psychology.* Beijing: Beijing Normal University Press.

